# Individual Differences in the Ability to Recognise Facial Identity Are Associated with Social Anxiety

**DOI:** 10.1371/journal.pone.0028800

**Published:** 2011-12-14

**Authors:** Joshua M. Davis, Elinor McKone, Hugh Dennett, Kirsty B. O'Connor, Richard O'Kearney, Romina Palermo

**Affiliations:** 1 The Department of Psychology, Australian National University, Canberra, Australian Capital Territory, Australia; 2 Australian Research Council Centre of Excellence in Cognition and Its Disorders (CCD), Perth, Western Australia, Australia; University of Adelaide, Australia

## Abstract

Previous research has been concerned with the relationship between social anxiety and the recognition of face expression but the question of whether there is a relationship between social anxiety and the recognition of face identity has been neglected. Here, we report the first evidence that social anxiety is associated with recognition of face *identity*, across the population range of individual differences in recognition abilities. Results showed poorer face identity recognition (on the *Cambridge Face Memory Test*) was correlated with a small but significant increase in social anxiety (*Social Interaction Anxiety Scale*) but not general anxiety (*State-Trait Anxiety Inventory*). The correlation was also independent of general visual memory (*Cambridge Car Memory Test*) and IQ. Theoretically, the correlation could arise because correct identification of people, typically achieved via faces, is important for successful social interactions, extending evidence that individuals with clinical-level deficits in face identity recognition (prosopagnosia) often report social stress due to their inability to recognise others. Equally, the relationship could arise if social anxiety causes reduced exposure or attention to people's faces, and thus to poor development of face recognition mechanisms.

## Introduction

Social anxiety is characterized by an intense concern about the impression one makes on others and represents anxiety situationally bound to social contexts [1], [2]. Socially anxious people fear they will behave in an unacceptable manner in social situations, and fear this may result in social rejection. While there are clinical forms of social anxiety (social anxiety disorder, social phobia [3]), social anxiety is also present, and varies in the healthy adult population.

It is well established that social anxiety affects facial *expression* processing (see reviews [4]–[6]) with an initial hypervigilance for threat followed by avoidance [7]. Compared to controls, patients with social phobia are faster at detecting angry than happy faces in visual search tasks [8] and have a bias to recognise faces with negative expressions [9] and those that they had previously categorized as critical rather than accepting [10]. However, people with high, but not clinical, levels of social anxiety do not appear to remember threatening faces more than those with low social anxiety [6].

Facial identity and expression differ in a number of ways. Expressions are changeable whereas identity is invariant, and cognitive and anatomical models of face processing argue that identity and expression processing are at least partially independent in the visual stream [11], [12] (see [13] for recent review). Given this independence, we examine here, for the first time, whether the recognition of facial *identity* is associated with variation in levels of social anxiety.

Such a relationship is theoretically plausible. Faces provide one of the primary means of discriminating between people, and the ability to recognise identity from the face facilitates social interactions. Individuals with clinical-level deficits of face identity recognition – that is, people with *prosopagnosia*, who find it very difficult to recognise faces including those of close friends and family – have described the inability to identify others as a constant source of social stress. In formal interviews [14], many people with developmental prosopagnosia described anxiety about social situations at work and at home, and how, because they could not recognise others, they avoided social gatherings or became dependent on close friends to help them through social interactions [14]. Anecdotal reports from single cases include “I think people think I'm ignoring them and rude when I walk past them in the corridor. They may say hello and I have no idea who they are” [15]; “It [prosopagnosia] makes me less interested in the social events, the partying, the getting to know lots of new people, because that just gives me a whole set of things I'll get wrong.” [14] (p. 448) and; “This condition always affects my ability to form normal social links to others. I prefer to be a recluse because I can't confidently function any other way. My avoidance of people (to interact with socially) is nearly phobic” [16] (p. 250).

The reports concerning prosopagnosia show that *extremely* poor face recognition can lead to social stress. Logically, it is also possible that distressing psychosocial consequences could be associated with milder “deficits” in face recognition ability [17]. It has recently been recognised that, far from everyone being “face experts”, there are large, reliable individual differences in face recognition abilities in the normal population (e.g. [18]–[20]). The present study is the first assessing whether these normal-range individual differences in facial *identity* recognition are associated with differences in social anxiety.

Two earlier studies have examined the relationship between face identity recognition and anxiety but have used measures of *general* anxiety. General anxiety assesses such things as general nervousness “I feel nervous and restless”, and coping with difficulties “I feel that difficulties are piling up so that I cannot overcome them” [21]. One of the studies [22] compared groups who were low and high on general anxiety (assessed using the Test Anxiety Scale; [23]), and reported better face recognition ability for the group low in general anxiety. The other [24] used the state and trait scales of the State-Trait Anxiety Inventory (STAI; [21]), and reported that, in females, trait anxiety and overall anxiety (sum of trait and state) were significantly correlated with face recognition, but there were no significant correlations in males (albeit with a smaller male sample size of only *n* = 29).

A potential problem with the general anxiety studies is that the stimuli contained not only face information but also hair and/or non-head information such as shoulders including clothing. It is well established that participants can use such information in laboratory tasks to recognise images, rather than using natural face recognition skills (e.g. [25]). However, hair and clothing provide only unreliable cues to identity in everyday life (e.g., a new shirt or a haircut would render a person unrecognisable). Thus, in the present study we employed a test that displays face information only, specifically the Cambridge Face Memory Test (CFMT; [26]). In this test, participants learn six neutral-expression target individuals, by viewing each face sequentially in three different views. In the test phase, participants choose the target face from two similar distractors. The target faces shown in the test phase are (a) images identical to those learned (Learn phase), (b) images that differ from those learned due to variations in viewpoint and lighting (Novel phase), or (c) images that differ from those learned due to variations in viewpoint and lighting and with visual noise added (Novel Images with Noise phase). The CFMT is a well-established test of face recognition and is known to show large and reliable differences in ability across the typical adult population [18], [19]. Our study tested the typical, non help-seeking, population. We assessed social anxiety with the Social Interaction Anxiety Scale (SIAS; [27]), which assesses anxiety in interpersonal and social situations. Sample items are “I find it difficult mixing comfortably with the people I work with” and “I worry about expressing myself in case I appear awkward”. A subset of participants also completed the trait component of the general anxiety State-Trait Anxiety Inventory (STAI-T; [21]), to determine whether any relationship is specific to social anxiety. To assess specificity of any relationship to faces, we assessed non-face object recognition ability in a subset of participants. The task was closely matched to the CFMT, except that it employed cars rather than faces (Cambridge Car Memory Test, CCMT; [28]). Finally, some participants completed Cattell's Culture Fair Intelligence Test Scale 3 (CFIT III; [29]), to examine the association between face identity recognition and nonverbal IQ.

## Methods

### Ethics Statement

All participants provided written informed consent to take part in this study. The research was approved by the Australian National University Human Research Ethics Committee (Protocol: 2009/274. Title: How humans perceive, recognise and evaluate visual images. Approved by the Chair of the Science/Med DERC on 16/06/2009).

### Participants and Design

The data derived from three separate studies. The tests of primary interest - the face recognition measure (CFMT) and the social anxiety scale (SIAS) - were included in all three. Other tests were included only in a subset of studies. The total combined sample comprised 138 (54 males) Caucasian adults aged 18 to 36 (*M* = 21.91, *SD* = 4.05 years). Participants were university students or others recruited via the Australian National University. They were unselected for face recognition ability, social anxiety level, or any other variable. Participants received $10 per hour, or completed the experiment as a part of university coursework. Note mean IQ for participants for whom this variable was measured (*N* = 63) was above average (see Table 1), and the range indicates our correlational results for IQ refer only to the upper half of the IQ distribution.

**Table 1:** Descriptive statistics for demographic variables and task performance.

	*N*	*M*	*SD*	Observed Range		Skewness	
				Min	Max	Statistic	*SE*
Age (years)	137	21.92	4.06	18	36	1.612	.207
Nonverbal IQ	63	122.46	11.82	94	152	−.122	.302
SIAS	137	22.61	12.69	1	60	.877	.207
STAI-T	78	40.74	10.92	23	70	.584	.272
CFMT-total	137	56.12	9.05	31	72	−.364	.207
CFMT-novel	137	22.75	4.88	11	30	−.402	.207
CCMT	70	52.69	9.12	24	70	−.447	.287

Note: Scale ranges are: SIAS = 0 (least anxious) to 80; STAI-T = 20 (least anxious) to 80; CFMT-total and CCMT = 24 (chance) to 72 (100% correct); CFMT-novel = 10 (chance) to 30 (100% correct).

In Study 1 (Davis, O'Connor, & Palermo; *N* = 66, 24 male), participants completed CFMT, SIAS, general trait anxiety measure (STAI-T), and nonverbal IQ (CFIT III) as part of a series of tests over a two-hour period. Responses to a questionnaire were used to exclude additional participants who reported: current clinical diagnosis of a mood or anxiety disorder; brain-related developmental disorder; head injury leading to more than two minutes of unconsciousness; or head injury resulting in ongoing effects such as amnesia. Some participants were tested in tutorial groups of up to 18 students (*n* = 37, 12 male); the remainder were tested individually (*n* = 29).

In Study 2 (Dennett, McKone, & Palermo; *N* = 56, 25 male), participants completed the CFMT as part of an initial 1-hour session and completed the SIAS and car task (CCMT) in a subsequent 1-hour session approximately one day later. All were tested individually.

In Study 3 (Davis, Dennett, Palermo, & McKone; *N* = 16, 5 male), participants completed SIAS, CFMT, STAI-T and CCMT in a single session. All were tested individually.

There were no significant differences in CFMT scores between the three studies, on either mean [Study 1: *M* = 56.39, Study 2: *M* = 55.46, Study 3: *M* = 56.44; *F*(2, 135) = .175], or variance [Study 1: *SD* = 9.03, Study 2: *SD* = 8.95, Study 3: *SD* = 10.43; Levene's test of equality of error variances, *F*(2,135) = .623]. Therefore, all three studies were collapsed and treated as a single sample for subsequent analyses.

### Test of facial identity recognition – Cambridge Face Memory Test (CFMT)

The CFMT ([26]; all upright faces) followed the standard instructions. The CFMT requires learning six target faces, each in three views. All faces are neutral expression.

In the *Learn* phase, the first target is presented in consecutive views (1/3-profile left, front, 1/3-profile right) for 3000 ms each. The next three trials show this target, in images identical to the study images, together with two distractors. The participant chooses the target by pressing a key (i.e. 3AFC task). This process is repeated for the other 5 target faces (to give 18 trials, i.e., 6 targets×3 views).

In the *Novel images stage* (30 trials), target faces are presented in previously unseen viewpoints and with different lighting conditions. Each test trial again presents three faces: the target (which can now be any one of the 6 learned individuals) and two distractor faces matched to the target for viewpoint and lighting. Each target is tested five times, once in each of five viewpoint/lighting conditions (front half-lit; 1/3-profile right, half shadow; 2/3-profile left; 2/3-profile right; and bottom-lit front). A given target does not appear in more than two consecutive trials. The same order is used for each participant.

The *Novel-images-with-noise* (‘noise’ stage; 24 trials) uses another new set of images (half-lit front; 1/3-profile left; 2/3-profile right; front facing). The task is made more difficult by adding coloured Gaussian noise to the faces, which alters the apparent shape and appearance of individual face features such as the nose or mouth. Each target is tested four times. Procedure is otherwise as for Novel stage.

Scoring is number of items correct out of 72 for CFMT-total. We also examined scores separately for CFMT-novel (out of 30) and CFMT-noise (out of 24). CFMT-Learn is not of interest in individual differences studies because typically-developing participants score at ceiling in this section [26].

The CFMT has been demonstrated to have good validity (low or no correlations with non-face identity tasks; ability to diagnose prosopagnosia; large inversion effects) and high reliability (e.g. [18], [19], [26], [30]). For an Australian population, internal consistency is Cronbach's alpha = .89 for CFMT-total [18].

### Social anxiety measure – Social Interaction Anxiety Scale (SIAS)

Participants completed the SIAS [27] in hardcopy. Participants rate on a five-point scale the extent to which they feel each statement is characteristic or true of them. Total scores on the 20-item scale range from 0 to 80, where higher scores indicate greater social anxiety. The SIAS exhibits high internal reliability, with Cronbach's alpha = .94 [27], as well as good discriminant and construct validity [31], [32].

### General anxiety – Trait scale from the State-Trait Anxiety Inventory (STAI-T)

The STAI-Trait scale (STAI-T; [21]) is a 20-item measure on which individuals rate on a 4-point scale the extent to which they “generally feel” various symptoms of anxiety. Total scores on STAI-T range from 20 to 80, where higher scores indicate greater anxiety. The STAI-T has good test-retest reliability, ranging from .82 to .94, and internal consistency ranging from .72 to .96 [33]. It also has well-established validity [21]. We assessed general anxiety via a trait, rather than state, measure because (a) [24] previously reported that only the trait anxiety correlated with their face-plus-hair-and-clothing recognition task, and (b) our social anxiety scale (SIAS) was also a trait measure.

### Car identification task – Cambridge Car Memory Task (CCMT)

The CCMT [28] was developed and kindly provided by Brad Duchaine and Raka Tavashmi. It is identical in form to the CFMT. The test requires learning six cars, and has three stages, totaling 72 trials: the learn stage (same images; 18 trials), the novel images stage (30 trials) and the novel images with noise stage (24 trials). The latter two stages involve recognition of the target cars over viewpoint and lighting changes. Total score out of 72 was calculated for each participant. The CCMT has shown high internal reliability, with Cronbach's alpha = .84 [28]. We selected the CCMT as our measure of non-face visual memory ability because it is well-matched to the CFMT in both the general cognitive requirements (memory, concentration, etc) and general perceptual requirements (i.e., within-class discrimination involving recognition across view/lighting change).

### Nonverbal IQ measure – Cattell's Culture Fair Intelligence Test Scale 3 (CFIT III)

The CFIT [29] provides a measure of nonverbal fluid intelligence [34]. Scale 3, Form A was used as it was designed for use with high-ability adults (suitable for our primarily university sample) and has high reliability, Cronbach's alpha = .74. It is a series of 50 geometric reasoning items, divided into four sections, each with a time limit for completion. Participants select two out of five possible responses in one test and one of several responses in the three other tests. The CFIT was administered as per the standard instructions in the test manual, which included practice questions.

### Data Screening

One case was removed as a multivariate outlier (Mahalanobis distance of Chi-square = 22.90, p<.001; score 17 on the SIAS compared to sample mean *M* = 22.81, *SD* = 12.67, and score 8 items correct on CFMT-novel compared to sample mean *M* = 22.68, *SD* = 4.88).

## Results

### Suitability of Distributions for Correlational Analyses

Table 1 shows descriptive statistics for each measure. As needed for correlational analysis, all showed a wide range of scores (see total range and *SD*).

Our anxiety measures showed non-normal distributions with positive skew. That is, an upper tail contained fewer individuals with high anxiety scores, while more participants had low-to-moderate scores. The SIAS showed significant skew (skew statistic = 0.877, *Z* = 4.24, *p*<.001) and a highly-significant overall deviation from normality (Shapiro-Wilk *W* = .941 (137), *p*<.001). The STAI-T also showed both skew (skew statistic = .584, *Z* = 2.15, *p* = .016) and deviation from normality (STAI-T: *W* = .954 (78), *p* = .007). There were also minor departures from normality on two other measures (CFMT-total: *W* = .974 (137), *p* = .011, CFMT-novel: *W* = .958 (137), *p*<.001), although this did not reflect significant skew (Table 1). The CCMT was normally distributed (*W* = .977 (70), *p* = .227), as was IQ (*W* = .984 (63), *p* = .593).

Given lack of normality, most subsequent analyses used Kendall's tau b (τ_B_) and Spearman's rho (ρ), non-parametric statistics used to measure strength of correlation, that do not rely on any assumptions about the distributions of variables. Spearman's ρ is a measure of average quadrant dependence (dependence between variables), while Kendall's τ_B_ is a measure of average likelihood ratio dependence (association between variables; [35]). Both τ_B_ and ρ may range from 1 to −1, where 1 indicates perfect correlation, and −1 indicates a perfect inverse correlation. A value of 0 indicates no correlation for both τ_B_ and ρ.

Some analyses also used an alternative approach to dealing with the skewed SIAS distribution, namely transforming the SIAS data by taking the square root of SIAS scores (sqrt-SIAS). This removed the non-normality (Shapiro-Wilk *W* = .991 (137), *p* = .450; Skew statistic = 0.134, *Z* = 0.647, *p* = .258), allowing standard Pearson's r, and multiple regression, to be used.

### Relationship between Face Identity Recognition and Social Anxiety

**Figure 1:**
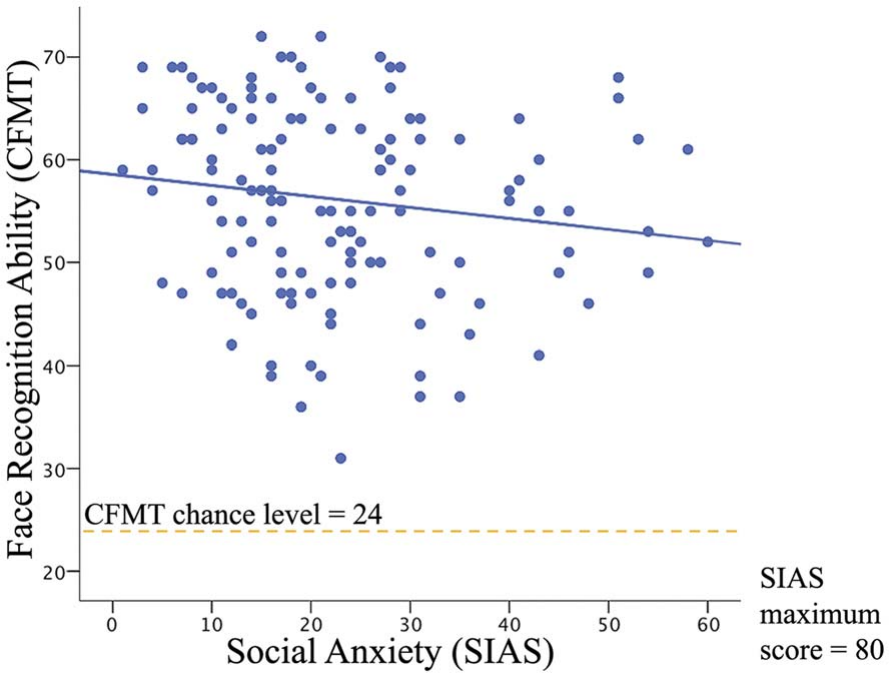
Scatterplot showing the negative correlation between face recognition ability (CFMT) and Social Anxiety (SIAS), indicating decreased face recognition ability is associated with increased social anxiety.

Using the full sample (i.e., full range of face recognition abilities present in an unselected population), analyses revealed a significant negative correlation between scores on the SIAS and scores on the CFMT-total (τ_B_ = −.128, *p* = .031; ρ = −.188, *p* = .028; *N* = 137; Figure 1). The correlation was also present using the sqrt-SIAS transformed scores (*r* = −.177. *p* = .039, *N* = 137). The relationship is in the predicted direction: that is, the negative correlation implies that *poorer* face recognition is associated with *higher* anxiety. The relationship with the SIAS was numerically strongest with the Novel stage of the CMFT (CFMT-novel), that is, the stage where the participants first have to recognise the target faces across views and lighting change, and performance in the typical population falls below ceiling (τ_B_ = −.159, *p* = .008; ρ = −.231, *p* = .007; *N* = 137; for sqrt-SIAS, *r* = −.226, *p* = .008). Note that the correlations, while significant, are small. The upper bound correlation (for a parametric Pearson's r) for the relationship between CFMT-Total and SIAS was .78 (calculated as the product of the square roots of the internal reliabilities for each task), much larger than the observed relationship of approximately .13–.19. Thus, as would be expected, there are many sources of variance in SIAS scores other than face recognition (and vice versa).

Note that we used the traditional p<.05 criterion for significance rather than correcting for the multiple correlations (involving other variables) reported in later sections, because (a) the key correlation between CFMT and SIAS was of *a priori* (not merely post hoc) interest to the study, and under these circumstances most researchers consider it acceptable to use p<.05; and (b) the correlations involving other variables reported later did not reach significance even uncorrected, so correcting would not change these findings (indeed, it would *decrease* confidence in the *non*significant relationships by decreasing power).

One possible caveat on the analyses above is that, because our participants were taken from a population unselected for face recognition ability, the sample could perhaps include some individuals who have developmental prosopagnosia. Prevalence estimates for developmental prosopagnosia are approximately 2–2.9% of the population [18], [36]. Given that our interest in the present study was specifically whether *normal*-range face recognition abilities are associated with social anxiety, it was important to address whether the associations between CFMT and SIAS reported above derive purely from the inclusion of any prosopagnosics in the sample. Although prosopagnosia can only be confirmed reliably via converging evidence from multiple sources – including Famous Face Tests and reports of relevant difficulties in everyday life such as trouble following films – CFMT performance that is 2 *SD*s below the mean is often indicative of the disorder (e.g., [18], [26]). Thus, to be cautious, we re-analysed the association between CFMT and SIAS removing the four individuals who had face recognition scores more than 2 *SD*s below the mean (cutoff score of 38.2 items correct for CFMT-total, using control norms from *N* = 248 young adult Australians; [15]). Evidence of a relationship was still present. The correlation between the CFMT-novel and SIAS was significant when these individuals were removed (τ_B_ = −.146, *p* = .017; ρ = −.212, *p* = .014; for sqrt-SIAS, *r* = −.195, *p* = .025, *n* = 133), and that between CFMT-total and SIAS approached significance (τ_B_ = −.114, *p* = .058; ρ = −.167, *p* = .054; for sqrt-SIAS, *r* = −.216, *p* = .012, *n* = 133).

### Relationships with general anxiety, IQ, and non-face object memory

An important question is whether the relationship between face identity recognition ability (CFMT) and social anxiety (SIAS) was independent of other factors. Results suggested it was.

First, the correlation appeared specific to social, rather than general, anxiety. There was no correlation between the CFMT-total and the STAI-T (τ_B_ = −.048, *p* = .548; ρ = −.068, *p* = .551 *n* = 78), nor between the CFMT-novel and the STAI-T (τ_B_ = −.071, *p* = .379; ρ = −.103, *p* = .371 *n* = 78). Note these correlations were not only non-significant, but also numerically extremely small. This was despite the moderate, significant correlation between the two anxiety measures themselves (SIAS with STAI-T, τ_B_ = .539, *p*<.001; ρ = .710, *p*<.001, *n* = 78). Given that it was previously found [24] that the STAI-T correlated with face-plus-hair-and-clothing recognition only in females, we also examined the correlations for each sex independently. With a face-only recognition task (CFMT) results failed to replicate previous findings of [24]: that is, for females, correlations were extremely small as well as non-significant between STAI-T and face recognition (CFMT-total, τ_B_ = −.033, *p* = .744; ρ = −.042, *p* = .773, *n* = 50; CFMT-novel τ_B_ = −.062, *p* = .539; ρ = −.080, *p* = .580, *n* = 50 here; cf *n* = 64 females in [24]). There was also no correlation for males (CFMT-total, τ_B_ = −.060, *p* = .662; ρ = −.079, *p* = .690, *n* = 28; CFMT-novel, τ_B_ = −.099, *p* = .474; ρ = −.139, *p* = .481, *n* = 28).

Second, concerning general cognitive abilities, the link between the SIAS and CFMT cannot be attributed to any relationships involving nonverbal IQ (as measured by CFIT III). Specifically, IQ was not correlated with either the CFMT (*r* = −.077, *p* = .550; τ_B_ = −.117, *p* = .194; ρ = −.153, *p* = .233, *n* = 63) nor the SIAS (τ_B_  = −.060, *p* = .501, *n* = 63; ρ = −.092, *p* = .474 *n* = 63). Also note that the direction of the very small trend between IQ and CFMT is negative, the reverse to that predicted if a relationship was present (which would be positive, i.e., higher IQ associated with higher face recognition scores).

Finally, concerning general object recognition ability, there was no significant correlation between the car recognition task and social anxiety (CCMT and SIAS, τ_B_ = −.107, *p* = .200; ρ = −.152, *p* = .290, *n* = 70). Note that although this correlation was not significant, it appears at first glance not much weaker numerically than the significant correlation between the CFMT and the SIAS (which was τ_B_ = −.128, *p* = .031; ρ = −.188, *p* = .028; *N* = 137). However, the subset of participants who completed the CCMT (*n* = 70 of 137) displayed a somewhat stronger correlation between the CFMT and the SIAS (τb = −.186, *p* = .026; ρ = −.273, *p* = .022, *n* = 70), making the distinction between the face and car correlations more apparent. To ensure that the correlation between face recognition and social anxiety could not be attributed to a general relationship present for all types of visual object recognition, we also conducted multiple regression using CFMT as the dependent measure, and entering CCMT and sqrt-SIAS as independent variables. Results showed a significant unique correlation between CMFT and sqrt-SIAS (semi-partial correlation = −.247, *p* = .039). This demonstrates a relationship between specifically *face* recognition and social anxiety.

Overall, these analyses argue that the relationship found between face recognition and social anxiety is not attributable to relationships with general anxiety, general cognitive ability, or general visual memory.

### A possible effect of sex?

One question is whether the correlation between face recognition and social anxiety is of equal magnitude in males and females. With *N* = 137 participants in total (54 male), our study did not have the statistical power to reliably test for a sex difference in the strength of the relationship. Our data contained some suggestion that the CFMT-SIAS correlation might be stronger in males (τ_B_ = −.198, *p* = .038; ρ = −.299, *p* = .028; *n* = 54, for CFMT-Total) than in females (τ_B_ = −.079, *p* = .304; ρ = −.116, *p* = .296; *n* = 83). However, the difference between the male and female correlations was far from significant (comparison on ρ values, *Z* = 1.07, *p* = .285 for CFMT-Total). Indeed, for our observed ρ values of −.299 (male) versus −.116 (female) to differ significantly at *p*<.05 would require a total sample size of *N* = 424 (212 men, 212 women). Overall, the current study is inconclusive as to whether there are sex differences in CFMT-SIAS relationship.

## Discussion

People with prosopagnosia often report that their inability to adequately recognise the faces of friends, family and colleagues is associated with social stress [14]. Here, our primary new finding is that, even within the normal range of face recognition abilities, there is also a small but significant relationship between face recognition ability and social anxiety, such that poorer face recognition skills are associated with higher social anxiety.

This association appeared to be specific to face recognition and to social anxiety. We did not observe a relationship between social anxiety and non-face visual memory (CCMT), indicating a specific relationship to faces rather than visual images in general. Note the face task and car task had similar means and SDs (see CFMT-total and CCMT in Table 1), so the presence of association only with the face task cannot be attributed to task difficulty differences or reduced variance in car task scores. We also found no association between face recognition and general, rather than social, anxiety. General anxiety and social anxiety are related, yet independent, constructs. Theoretically, we do not find it surprising that face recognition ability could be more strongly associated with the latter. Only social anxiety directly taps unease about interacting with people in social situations, the circumstance in which face recognition is typically needed to ensure reliable person identification.

A possible caveat to our general anxiety result is that only Study 1 and 3 participants completed the general anxiety measure. This subset of participants showed not only no correlation between general anxiety (STAI-T) and face recognition, but also a trend towards a weaker correlation than the full sample between social anxiety (SIAS) and face recognition. The reason for this is unclear, but it might possibly be related to the fact that this subset contained a slightly higher proportion of females (65%) than the full sample (61%), and a noticeably higher proportion of group-tested participants (45%) than the full sample (27%). Also our data showed (non-significant) trends for the correlation between CFMT and SIAS to be weaker in these groups than in men and in individually-tested participants.

We also cannot rule out the possibility that general anxiety may play some mediating role in affecting the strength of relationship between face recognition and social anxiety, and it would be valuable for future studies to investigate this possibility. Our lack of association between face recognition and general anxiety might appear to contrast with two earlier studies reporting a relationship between “face” recognition and general anxiety, at least in females [22], [24]. However, as noted earlier, their stimuli contained not only faces but also hair and clothing. Thus, the results of the present study are not in direct conflict with the previous findings. Instead, general anxiety may be related to visual memory performance only when non-face cues are present and deliberate strategic processing is valuable in assisting performance, such as via implementation of memory strategies (e.g., verbal rehearsal of “wide necktie, wide necktie”).

Our final finding was that face-only recognition was independent of general cognitive abilities (non-verbal IQ, i.e., CFIT-III). This in agreement with several previous studies, showing no or very weak correlation of face memory with nonverbal reasoning (Raven's advanced progressive matrices, [20]), the WAIS-R(S) [37], and verbal memory (single words, [18]; paired-associate learning, [19]). Thus, there is now strong evidence that face recognition is independent of intelligence, at least in the upper half of the IQ distribution (there is little data available on the lower half).

One question left open by our present study is whether there is any sex difference in the strength of the relationship between face recognition and social anxiety. Our results suggest the correlation between CFMT and SIAS may possibly be stronger in males than in females, but also that it would take a very large sample size, three times that of the current study, to be able to address this question reliably. Note our direction of trend for *social* anxiety is the opposite to the trend reported by [24] for the correlation between *general* anxiety and face-plus-hair-and-clothing recognition (which was stronger in females than in males).

In sum, the results demonstrate, for the first time, a relationship between face identity recognition and social anxiety. This relationship was statistically significant, but it is also important to note that it was rather small. This is unsurprising: social anxiety is a complex construct, which is dynamic across situations [32], and so theoretically we would expect the relationship between social anxiety and any single variable to be relatively weak. Practically, the weak correlation also implies that, in future studies, further support for an association between social anxiety and face identity recognition might best be obtained and investigated via studies with very large samples (e.g., see [38] for a study where a very large sample, n = 4608, was able to show a strong relationship between emotional experience and emotion recognition).

### Possible mechanisms underlying the correlation between face recognition and social anxiety

We now consider the issue of causation. Importantly, the relationship we observed is merely a correlation, and thus does not necessarily indicate a *direct* causal relationship, of any form, between face recognition and social anxiety (e.g., both could be caused by a third, unmeasured, variable). Further, even if there is a direct link, our results do not tell us which direction this causality would take. However, as we argue below, both directions of bi-variate causation would in fact be theoretically plausible, thus making it valuable for future studies to test for direct causal relationships (e.g., by providing interventions for social anxiety and observing any effects on face recognition performance; or by training face recognition and observing any effects on social anxiety).

First, we consider the hypothesis that *poor face recognition leads to social anxiety*. The plausibility of this idea comes primarily from the self-reports of individuals with prosopagnosia. Many of these reports make it clear that not only do many suffer social stress, but that they attribute this directly to their inability to identify other people, particularly in large group settings, or where a person is met out of context (e.g. [14]). In fact, it has been proposed [14] that developmental prosopagnosia is a risk factor for the development of certain aspects of social anxiety disorder (those pertaining to anxiety about social interaction rather than performance). As noted in [14], the risk of social anxiety disorder is likely to be mediated by personality and social circumstances. It is also of interest to note that a tendency to withdraw from social situations appears to precede the development of anxiety disorders [39]. Difficulty recognising faces may be one reason to avoid social situations.

Second, regarding the hypothesis that *higher social anxiety leads to poorer face recognition*, a plausible chain of causality in this direction can also be constructed. This is that (a) high social anxiety causes less exposure to faces (because individuals choose to interact with fewer others) and/or lack of appropriate attention to faces (because individuals concentrating on their own anxiety in the social setting may pay less attention to the faces of others, even when others are present, e.g., see [4]) and/or the appropriate parts of a face, such as the eyes (e.g., [40] for data with social phobics); and then (b) this lack of exposure/attention, especially over a prolonged period or in the course of childhood, leads to poor development of perceptual face processing skills needed to distinguish individuals. The idea that lack of attention to faces could lead to face recognition difficulties has been proposed in Autism Spectrum Disorder (ASD) [41]–[43].

Finally, causality may be present that is *bi-directional* across development. Given that it seems equally plausible that poor face recognition could lead to social anxiety, and that social anxiety could lead to poor face recognition, it is possible that both of these factors operate across the course of childhood. For example, a young child with initially poor face recognition might find social interaction more difficult than other children, leading to the beginnings of social anxiety, which in turn leads to avoidance of social situations and/or lack of attention to faces, which leads to failure to show normal developmental improvement in face recognition, which leads back to increased social anxiety, and so on (see [44] for a discussion of causal modeling in developmental disorders).

### Conclusion

Despite the traditional focus on face expression in psychosocial research, it is more recently becoming clear that face identity recognition is also important. Previous individual differences studies have shown that identity recognition is associated with extraversion-intraversion [45], [46], and with empathy [47], and our present study has extended the relationship with social factors to social anxiety. Researchers and clinicians treating social anxiety have not traditionally considered that a basic perceptual skill like face recognition could be a contributing factor for social difficulties in some individuals. Our results suggest that it may be valuable to do so.
